# Comparison of the Magnesium Sulphate With Paracetamol Combination vs the Fentanyl With Lignocaine Combination in Attenuating the Hemodynamic Response During Laryngoscopy and Intubation: A Prospective, Double-Blinded Randomized Controlled Study

**DOI:** 10.7759/cureus.66241

**Published:** 2024-08-05

**Authors:** Nandhakumar Murugesan, Amoolya Kamalnath, R V Ranjan, Sivakumar Segaran

**Affiliations:** 1 Anaesthesiology, Muthus Ortho Hospital, Coimbatore, IND; 2 Anaesthesiology, Pondicherry Institute of Medical Sciences, Pondicherry, IND; 3 Anaesthesiology, Sri Manakula Vinayagar Medical College and Hospital, Pondicherry, IND

**Keywords:** intubation, laryngoscopy, lignocaine, fentanyl, paracetamol, magnesium sulphate, haemodynamic response

## Abstract

Background and aims

Laryngoscopy and intubation cause an increased sympatho-adrenergic pressor response, which can be detrimental to patients with coronary artery disease, hypertension, etc. Various drugs and manoeuvres have been tried to reduce the pressor response with acceptable results but the quest for the ideal drug still continues. Hence, we planned to compare the effects of magnesium sulfate with paracetamol and fentanyl with lignocaine on attenuating the hemodynamic responses due to direct laryngoscopy and intubation and to note the complications of these drugs.

Methods

We studied 60 adult patients of the American Society of Anaesthesiologists (ASA) physical status I and II of either sex, scheduled for elective surgery under general anaesthesia. The patients were randomly divided into two groups. Group A received 25 mg/kg magnesium sulphate mixed with paracetamol 1 gram IV (100 ml) given over 10 minutes before induction and Group B received 2 mcg/kg fentanyl and 1.5 mg/kg lignocaine, 3 minutes before intubation. All patients were uniformly pre-medicated, induced, and intubated as per standard protocol. Heart rate (HR) and systemic arterial pressures were recorded at baseline, after study drug infusion, after induction, and 1, 3, 5, 10, and 15 mins after intubation. Hemodynamic parameters were compared using repeated measures analysis of variance (ANOVA). In the post-hoc tests, p value < 0.05 was considered statistically significant.

Results

We observed the mean pre-op HR (p = 0.161) and mean HR one-minute post-induction (p = 0.144). The percentage change from baseline at one-minute post-induction was 9.7 in Group A and 15.2 in Group B. We observed the mean pre-op mean arterial pressure (MAP) (p = 0.119) and mean MAP one minute post-induction (p = 0.585). The percentage change from baseline at one-minute post-induction was 3.3 in Group A and 2.8 in Group B. The percentage change from baseline was found to be within 15%, for HR in Group A and for systolic blood pressure (SBP), diastolic blood pressure (DBP), and MAP in Group B. However, there was no statistically significant difference (p > 0.05) between the mean HR, SBP, DBP, and MAP between the time points.

Conclusion

In our study, both the combinations of drugs, magnesium sulphate with paracetamol (Group A drugs) and fentanyl with lignocaine (Group B drugs) were found to be equally effective (i.e. neither group was superior to the other) in attenuating the hemodynamic response to laryngoscopy and intubation.

## Introduction

Many patients undergoing surgery under general anesthesia (GA) require laryngoscopy and endotracheal intubation as mandatory procedures; hence, they become an integral part of GA. It has been found that laryngoscopy and intubation cause an increase in the serum concentration of catecholamines, leading to tachycardia, hypertension, and dysrhythmias. These changes can lead to complications like myocardial infarction and cerebrovascular accidents in susceptible individuals [1].

Administration of potent opioid analgesics like fentanyl can significantly control the increase in blood pressure (BP) and heart rate (HR) during laryngoscopy and intubation, particularly in neurosurgical patients [2]. Short-duration laryngoscopy combined with viscous and intravenous (IV) lidocaine just before tracheal intubation can effectively minimize the increase in mean arterial pressure (MAP) during endotracheal intubation and cause spontaneous reduction in BP and HR following the placement of an endotracheal tube [3].

Magnesium sulfate (MgSO4) inhibits the release of catecholamines from the adrenal medulla and is effective in attenuating the sympathetic response to surgical stimulus [4]. In addition, it has multiple effects: (i) anti-arrhythmic, (ii) potentiates the effects of analgesics, (iii) bronchodilator, and (iv) hypotensive. The dose of MgSO4 can be reduced if it is mixed with other potent analgesics like IV paracetamol. The main mechanism of action of paracetamol is the inhibition of the cyclo-oxygenase enzyme which is responsible for the production of prostaglandins, an important mediator of inflammation, pain, and fever [5].

Paracetamol and MgSO4 have been used to suppress pain as well as sympathetic response due to noxious stimuli during the peri-operative period. However, their combination to suppress laryngoscopic response has not been studied extensively. Hence, we aimed to evaluate the efficacy of MgSO4 with paracetamol in controlling the hemodynamic response during laryngoscopy and intubation. We hypothesize that by combining magnesium sulfate with paracetamol, we will be able to achieve the same effect as with fentanyl and lignocaine for the prevention of hemodynamic responses during laryngoscopy and intubation. Our primary objective was to compare the effects of combinations of MgSO4-paracetamol with fentanyl-lignocaine in attenuating hemodynamic responses to direct laryngoscopy and intubation and our secondary objective was to note the complications of these drugs.

## Materials and methods

A prospective, double-blinded randomized controlled study was designed. Institutional ethical committee clearance was obtained (IEC: RC/19/112, CTRI/2019/12/022315, 11/12/2019). The study period was from November 2019 to March 2021. Patients admitted to Pondicherry Institute of Medical Sciences who underwent elective surgeries under general anesthesia with patients’ ages being between 18 and 50 years, American Society of Anaesthesiologists (ASA) physical status I and II patients, and patients posted for elective surgeries under GA were included in the study. Patients in whom endotracheal intubation could not be achieved within 15 seconds by an experienced anaesthesiologist, patients with HR less than 50 per minute, systolic blood pressure (SBP) less than 100 millimeters of mercury (mmHg), presence of any degree of heart block, history of obstructive lung disease, patients on any cardiovascular medications, and patients with difficult airway were excluded from the study.

**Figure 1:**
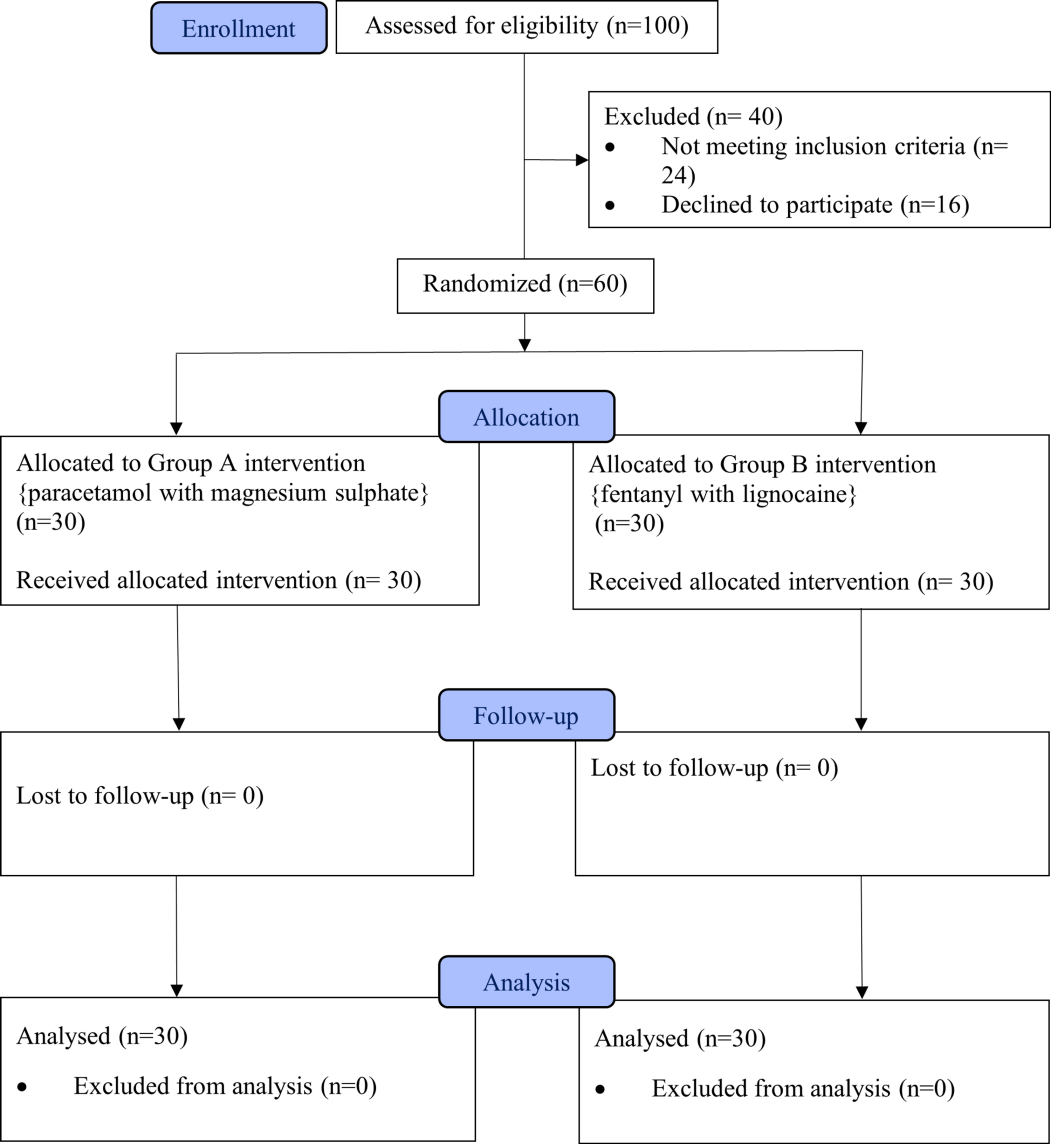
Consort diagram based on 2010 guidelines

A pre-anesthetic evaluation was done for all patients one day before surgery. Informed written consent was obtained from the patients. Patients were pre-medicated with tablet ranitidine 150 mg and tablet metoclopramide 10 mg at 6 a.m. on the day of the surgery. On arrival to the operating room, vascular access was obtained using an 18G cannula, and a 10 ml/kg/hour Ringer lactate infusion was started, 0.03 mg/kg IV midazolam was administered, and patients were connected to standard ASA monitors. The patients were randomized into two groups (A and B) with computer-generated random numbers (Figure 1).

The study drug was prepared by the consultant anesthesiologist (operator). The observer and the patients were blinded to the drug they received, and the operator administered the drugs 10 minutes before the induction of anesthesia. Group A received 25 mg/kg MgSO4 mixed with 1 gram of paracetamol (100 ml) infused over 10 minutes and Group B received 100 milliliters (ml) of normal saline over 10 minutes, both via a separate infusion set through a three-way connector at a rate of 10 ml/min. Three minutes before intubation, Group A received 10 ml of normal saline as a bolus and Group B received fentanyl two mcg/kg and lignocaine 1.5 mg/kg as a bolus mixed in a 10 ml syringe.

After pre-oxygenation with 100% oxygen, patients were induced with propofol two mg/kg, and vecuronium 0.1 mg/kg was used to facilitate endotracheal intubation. Patients were manually ventilated for three minutes after which, the same consultant anesthesiologist performed the laryngoscopy and intubation using a Macintosh laryngoscope, and an appropriate-sized cuffed endotracheal tube was inserted. After intubation, patients were maintained with isoflurane-50% oxygen-50% nitrous oxide to attain one minimum alveolar concentration (MAC), and injection vecuronium 0.025 mg/kg was given intermittently. Hemodynamic parameters like HR, SBP, diastolic blood pressure (DBP), and MAP were recorded at the following intervals: pre-operative, before induction, after induction, and 1, 3, five, 10, and 15 minutes after laryngoscopy and intubation. Those patients whose HR dropped more than 20% from baseline and whose SBP, DBP, and MAP dropped more than 20% from baseline were appropriately managed but excluded from the study. For an increase in HR of more than 100 per minute, injection esmolol 0.5 mg/kg IV; for a decrease in HR less than 50 per minute, injection atropine 0.6 mg IV, for a decrease in BP more than 20% from baseline, and injection ephedrine 6 mg IV, were given, and the patients were assessed. The patients were ventilated to maintain end-tidal carbon dioxide levels between 30 and 35 mmHg. A surgical incision was made following the completion of the data collection process.

At the end of the surgical procedure, the residual neuromuscular blockade was antagonized and routine awake extubation was performed. Patients were monitored in the recovery room for 60 minutes. Patients were observed postoperatively for 24 hours for any complications.

The demographic and clinical parameters of the patients were recorded. Descriptive statistics, such as mean and standard deviation (SD), were used for continuous variables, and number and percentage for categorical variables. Repeated measures analysis of variance (ANOVA) was used to compare hemodynamic parameters (HR, SBP, DBP, MAP) over time intervals. Further, post-hoc tests using Bonferroni correction were also done and any p value < 0.05 was considered statistically significant. The software used was IBM SPSS version 26.0 (IBM Corp., Armonk, NY, US).

Sample size calculation was done from a previous similar study comparing IV MgSO4 and IV lignocaine taking the difference in the HR variability [6]. A power analysis indicated that a minimum of 54 patients would be needed to reach 80% power with an alpha error of 0.05 to reject the null hypothesis. Considering the 10% dropout rate, a total of 60 patients were included (30 in each group).

## Results

All the parameters were found to follow the normal distribution. This was tested with the Shapiro-Wilk test. The age and gender distribution, ASA status, and body mass index (BMI) were comparable in both groups with no statistical significance between them (Table 1). The student’s unpaired t-test was used for age and BMI. The chi-square test was used for gender and ASA status.

**Table 1:** Demographic characteristics of participants ASA - American Society of Anaesthesiologists, BMI - Body Mass Index, SD - Standard Deviation

Group	Age		Gender			ASA			BMI	
			Male (n)	Female (n)		ASA 1	ASA 2			
	Mean (S.D)	P value			P value			P value	Mean (S.D)	P value
A	40.77 (12.938)	0.250	19	11	0.194	18	12	0.436	24.75 (3.535)	0.995
B	39.43 (14.039)		14	16		15	15		23.43 (3.990)	

**Figure 2:**
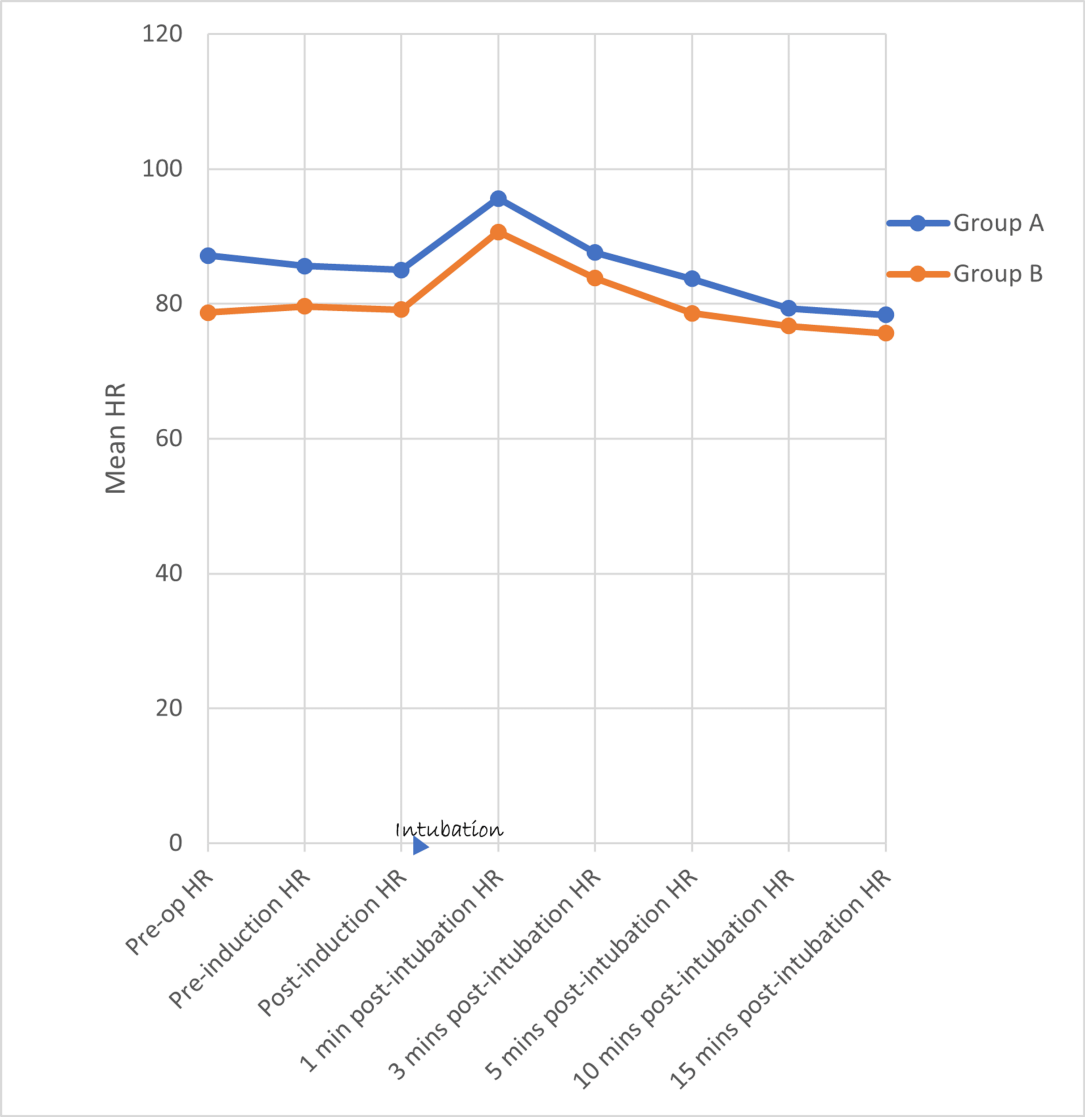
Distribution of mean heart rate at various time intervals between the two groups HR - Heart Rate, Min(s) - Minute(s)

**Figure 3:**
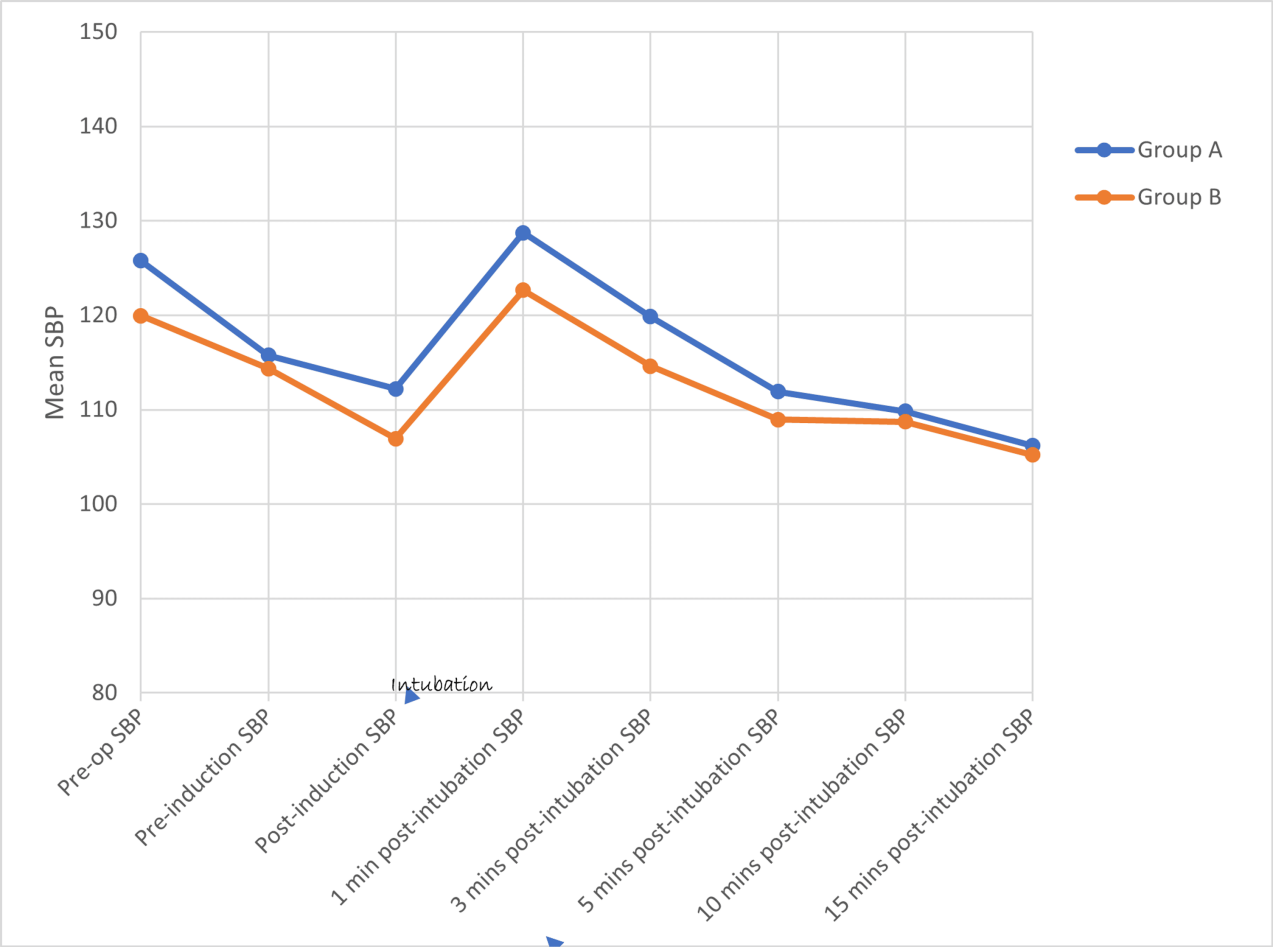
Distribution of mean systolic blood pressure at various time intervals between the two groups SBP - Systolic Blood Pressure, mmHg - Millimetres of Mercury, Min(s) - Minute(s)

**Figure 4:**
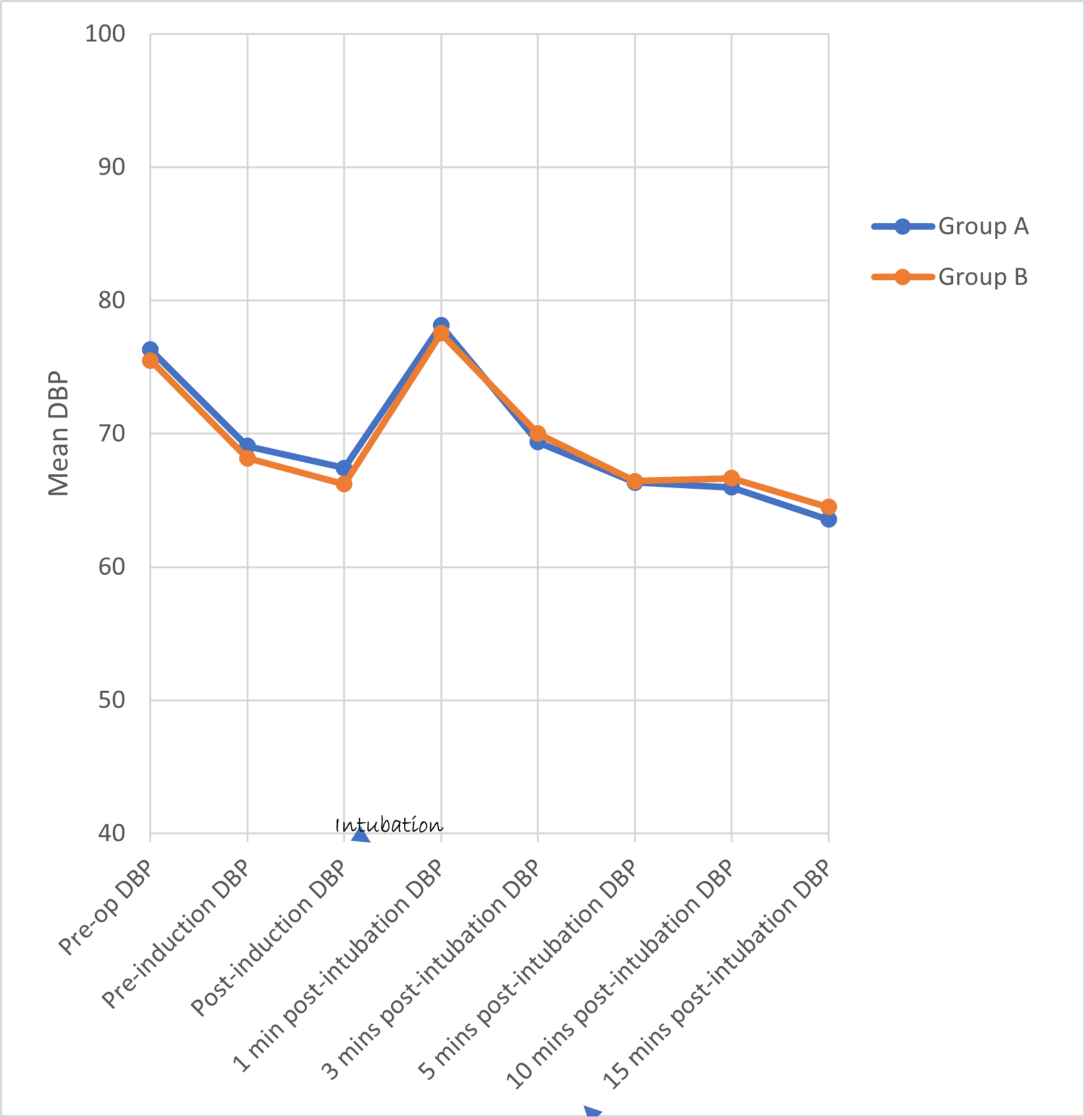
Distribution of mean diastolic blood pressure at various time intervals between the two groups DBP - Diastolic Blood Pressure, mmHg - Millimeters of Mercury, Min(s) - Minute(s)

**Figure 5:**
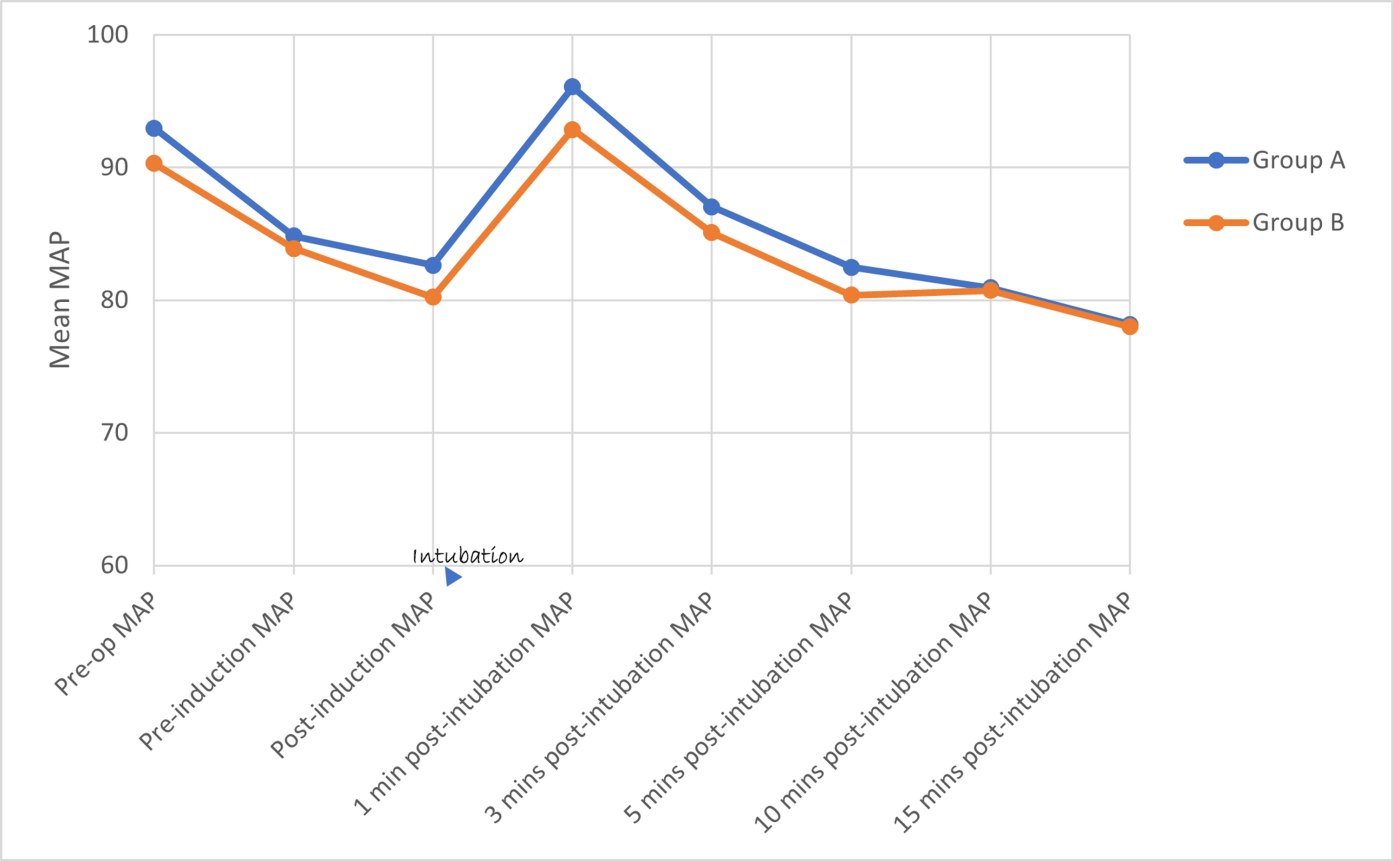
Distribution of mean values of mean arterial pressure at various time intervals between the two groups MAP - Mean Arterial Pressure, mmHg - Millimeters of Mercury, Min(s) - Minute(s)

Using the student’s unpaired ‘t’ test, there is no statistically significant difference in HR (Figure 2), SBP (Figure 3), DBP (Figure 4), and MAP (Figure 5) between both groups at various time intervals.

In Group A, a repeated measures ANOVA with a Greenhouse-Geisser correction determined that mean HR, SBP, DBP, and MAP did not differ significantly between time points (p value = 0.490, 0.426, 0.684, and 0.590, respectively). Post-hoc tests using the Bonferroni correction revealed that there is (a) a rise in HR at 1 and 3 minutes (b) a reduction in SBP at 3, 5, 10, and 15 minutes (c) a statistically significant reduction in DBP and MAP at 5, 10, and 15 minutes, following the Group A drug compared to pre-operative HR, SBP, DBP, and MAP, respectively. The maximum rise in HR, SBP, DBP, and MAP reached at one minute after intubation (9.7%, 2.4%, 2.4%, and 3.3%, respectively) following the Group A drug when compared to pre-operative levels but the rise was not statistically significant. There is a better reduction in (a) HR and SBP at 5, 10, and 15 minutes and (b) DBP and MAP at 3, 5, 10, and 15 minutes after intubation when compared to the baseline (pre-operative values).

In Group B, repeated measures ANOVA with a Greenhouse-Geisser correction determined that mean HR, SBP, DBP, and MAP did not differ significantly between time points (p value = 0.489, 0.498, 0.613, and 0.5777). Post-hoc tests using the Bonferroni correction revealed that there is (a) a rise in HR at 1 and 3 minutes, (b) a reduction in SBP at 3, 5, 10, and 15 minutes, (c) a statistically significant reduction in DBP at 15 minutes, (d) a statistically significant reduction in MAP at 5, 10, and 15 minutes following the Group A drug compared to pre-operative HR, SBP, DBP, and MAP. The maximum rise in HR, SBP, DBP, and MAP reached at one minute after intubation (15.7%, 2.3%, 2.7%, and 2.8%, respectively) following the Group B drug when compared to pre-operative HR, SBP, DBP, and MAP, but the rise is not statistically significant. There is a better reduction in SBP at 5, 10, and 15 minutes after intubation when compared to the baseline (pre-operative) SBP. There is a better reduction in DBP and MAP at 3, 5, 10, and 15 minutes after intubation when compared to the baseline (pre-operative) DBP and MAP.

The groupwise comparison of HR and MAP from baseline to various time intervals in Group A and Group B is provided in Table 2 and Table 3, respectively.

**Table 2:** Groupwise comparison of heart rate from baseline to various time intervals in Group A and Group B HR - Heart Rate, bpm - Beats Per Minute, Std. - Standard, Sig. - Significant, Pre-op - Pre-operative, min - Minute(s), I - Pre-operative Value of HR, J - Value of HR at 1, 3, 5, 10, and minutes

Group A	(I) HR (bpm)	(J) HR (bpm)	Mean Difference (I-J)	Std. Error	Sig. P Value
Paracetamol and magnesium sulfate	Pre-op	1 min	-8.433	4.091	0.612
		3 min	-0.4	4.091	1
		5 min	3.5	4.091	1
		10 min	7.833	4.091	0.858
Group B	(I) HR (bpm)	(J) HR (bpm)	Mean Difference (I-J)	Std. Error	Sig. P Value
Fentanyl and lignocaine	Pre-op	1 min	-11.967	2.913	0.001*
		3 min	-5.1	2.913	1
		5 min	0.067	2.913	1
		10 min	2	2.913	1
	*The mean difference is significant at the 0.05 level.				

**Table 3:** Groupwise comparison of mean arterial pressure from baseline to various time intervals in Group A and Group B MAP - Mean Arterial Pressure, mmHg - Millimeters of Mercury, Std. - Standard, Sig. - Significant, Pre-op - Pre-operative, min - Minute(s) I - Pre-operative Value of MAP, J - Value of MAP at 1, 3, 5, and 10 minutes

Group A	(I) MAP (mmHg)	(J) MAP (mmHg)	Mean Difference (I-J)	Std. Error	Sig.
					p Value
Paracetamol with magnesium sulfate	Pre-op	1 Min	-3.1	2.69	1
		3 Min	5.933	2.69	0.431
		5 Min	10.500*	2.69	0.002*
		10 Min	12.033*	2.69	0.000*
		15 Min	14.800*	2.69	0.000*
	*The mean difference is significant at the 0.05 level.				
Group B	(I) MAP (mmHg)	(J) MAP (mmHg)	Mean Difference (I-J)	Std. Error	Sig.
					p Value
Fentanyl and lignocaine	Pre-op	1 Min	-2.567	2.955	1
		3 Min	5.2	2.955	1
		5 Min	9.933*	2.955	0.014*
		10 Min	9.567*	2.955	0.022*
		15 Min	12.300*	2.955	0.001*
	*The mean difference is significant at the 0.05 level.				

## Discussion

Laryngoscopy and endotracheal intubation have been traditionally considered to be integral to conducting GA but they also provoke a reflex cardiovascular response, which is evoked by the stimulation of laryngeal and tracheal tissues during the procedure [7]. These changes are due to an increased sympatho-adrenergic pressor response and can lead to complications that are tolerated by healthy individuals but are detrimental in patients with co-morbidities [8-10]. The attenuation of this pressor response to laryngoscopy and intubation is one of the most researched topics in the field of anesthesiology. Many methods like limiting the duration of laryngoscopy to 15 seconds and the use of lignocaine, low-dose opioids (1-3 mcg/kg of fentanyl or alfentanil 80-100 mcg/kg, morphine 0.2 mg/kg), MgSO4, nitroglycerine, beta-blockers like esmolol, etc. [11-14] have been tried to reduce the pressor response.

Fentanyl, a synthetic narcotic analgesic with rapid onset and short duration of action, is very effective in attenuating cardiovascular, metabolic, and hormonal responses and inhibits catecholamine release during the stress response of laryngoscopy, endotracheal intubation, and surgical stimuli.

Administration of IV lignocaine, a local anesthetic that produces a reversible conduction blockade of impulses along central and peripheral pain pathways after regional anesthesia, inhibits arrhythmias, and suppresses the hemodynamic responses during laryngoscopy and intubation [15-17].

MgSO4 produces vasodilation by interfering with a wide range of vasoconstrictor substances and by directly acting on the smooth muscles of blood vessels. Due to its dual action of anti-arrhythmic effects and the ability to inhibit the release of catecholamines, along with calcium antagonist and vasodilator properties, it has been used to attenuate the hemodynamic stress response during laryngoscopy and endotracheal intubation.

Paracetamol may cause hypotension either by reducing SBP or MAP, and it can be administered through the IV route, which has peak plasma concentration within 10 minutes after the start of infusion. Recently, it has been tried to reduce the pressor response during laryngoscopy and intubation [18].

Comparing 30 mg/kg of MgSO_4_ and 1.5 mcg/kg of fentanyl, Saroj et al. [19] concluded that MgSO4 and fentanyl were equally good in attenuating the HR response to laryngoscopy and intubation (p > 0.05). At 1, 3, 5, 10, and 15 minutes after intubation, there was no statistically significant difference in HR in their study. We also observed that both MgSO4-paracetamol and fentanyl-lignocaine combinations equally attenuated the rise in HR after intubation.

In a study conducted by Padmawar et al. [6], the hemodynamic responses occurring after the administration of 40 mg/kg of 50 % MgSO4 and 1.5 mg/kg of 2% lignocaine were compared. In the MgSO4 group, SBP decreased after premedication and increased one minute after intubation, which was normalized at five minutes after intubation, but in the lignocaine group, the rise after intubation did not return to baseline even five minutes after intubation. In our study, we observed that both MgSO4-paracetamol and fentanyl-lignocaine combinations equally attenuated the rise in SBP after intubation and were also effective in controlling SBP after laryngoscopy and intubation.

The effect of 40 mg/kg of MgSO4 was compared with two mcg/kg of fentanyl and with the control group, in controlling the hemodynamic response during laryngoscopy and intubation [20]. Megalla et al. found that there was a significant increase in DBP after intubation in all three groups (p < 0.000). The maximum rise in DBP was in the first minute after intubation followed by a gradual decrease in all three groups over time. There was a statistically significant increase in DBP in the control group when compared with both fentanyl and MgSO4 (p < 0.000) groups. They concluded that both fentanyl and MgSO4 decreased the pressor response during laryngoscopy and intubation when compared to the control group, but fentanyl at the dose of 2 mcg/kg did not completely abolish the pressor response. This may be due to inadequate doses or inappropriate timing for fentanyl. However, in our study, we administered a combination of fentanyl with lignocaine at three minutes before intubation, which adequately attenuated the stress response. Hence, both the dose and the timing of the drug administered are equally important in attenuating the hemodynamic responses.

Nooraei et al. [21] compared the effect of 60 mg/kg of IV MgSO4 and 1.5 mg/kg of IV lignocaine in attenuating the hemodynamic response during laryngoscopy and tracheal intubation. Both groups had a rise in MAP after intubation but MAP increased significantly in the lignocaine group at one and two minutes after intubation and it came to near baseline at five minutes after intubation. However, in our study, MAP did not significantly increase after intubation at any time intervals; this is because we combined lignocaine with fentanyl, which attenuated hemodynamic responses better than lignocaine alone as given in their study. In fact, in our study, we observed that both MgSO4-paracetamol and fentanyl-lignocaine combinations equally attenuated the rise in MAP after intubation.

We used 25 mg/kg of MgSO4 while in all four of the above-mentioned studies, the dosage of MgSO4 used is higher. Our study proves that the desired effect of laryngoscopic response can still be achieved with 25 mg/kg by the addition of 1 gram of paracetamol to adequately minimize the stress responses while preventing the unwanted side effects of MgSO4 (like bradycardia and delayed recovery), which may occur with the higher dosages of the drug. All the above studies compared two individual drugs, whereas we compared two combinations of drugs. We observed that using a combination of drugs was more effective than using a single drug in attenuating the response to laryngoscopy and intubation.

Rare complications expected from these drugs are allergic reactions, arrhythmias, hypotension, and delayed recovery from anesthesia. However, our sample size was too small to comment on complications arising from these drugs. In their study, Czarnetzki et al. [22] found that the patients who were pre-treated with 60 mg/kg of MgSO4 complained of a burning sensation in the arm and heat sensation all over the body during drug administration, but none of these complaints occurred in the placebo group. In our study, none of the patients developed any side effects. This is because we used a combination of the drugs with minimal doses, which prevented the unwanted side effects of the drugs.

Strengths

The employment of the technique of randomization and the meticulous planning and administration of the combinations of drugs as mentioned in the methodology reduced the occurrence of bias in our study.

Limitations

Laryngoscopy and intubation were done by different anesthesiologists with varying experience. This leads to a change in the duration of laryngoscopy in each patient and an increase in sympatho-adrenal response. Invasive BP monitoring would have been more ideal for giving accurate BP readings, which was not used in our study. Neuromuscular monitoring would have given the exact idea of muscle relaxant effect augmentation by MgSO4, which we did not use.

## Conclusions

In this study, we compared the combinations of MgSO4-paracetamol and fentanyl-lignocaine for the attenuation of the hemodynamic response to laryngoscopy and endotracheal intubation in patients undergoing general anesthesia. Based on our results, we conclude that both combinations of drugs are equally effective (i.e. neither group was superior to the other) in the attenuation of hemodynamic responses to laryngoscopy and intubation. However, based on clinical observations of percentage change from the baseline, the combination of MgSO4-paracetamol better attenuates the heart rate response to laryngoscopy and intubation, and the combination of fentanyl-lignocaine better attenuates blood pressure response to laryngoscopy and intubation. Both the dose and the timing of the drugs administered are important. Side effects and complications from larger doses of the drugs were prevented in our study by using lower doses but in combination with a companion drug. This adequately minimized the stress responses.

A study with a larger sample size can be planned in the future using invasive BP monitoring and neuromuscular monitoring.
